# Optical coherence tomographic findings of ocular siderosis following intraocular foreign body removal: A case report

**DOI:** 10.1097/MD.0000000000021476

**Published:** 2020-07-24

**Authors:** You Hyun Lee, Yu Cheol Kim

**Affiliations:** Department of Ophthalmology, Keimyung University School of Medicine, Dongsan Hospital, Daegu, Korea.

**Keywords:** case report, intraocular foreign body, ocular siderosis, optical coherence tomography

## Abstract

**Rationale:**

Ocular siderosis is arrested by the removal of intraocular foreing body (IOFB). The progression of ocular siderosis is very rare and few reports demonstrate the optical coherence tomographic (OCT) findings.

**Patient concerns:**

A 55-year-old Asian man presented to our clinic with the chief complaint of decreased vision in his left eye for 5 months. On slit lamp examination of the left eye, the corneal stroma had a rust-colored hue, and the retina was not visible due to vitreous opacity. An orbital computed tomography was ordered considering the possibility of left IOFB, which confirmed the presence of a vitreous IOFB. On the next day, he had a continuous curvilinear capsulorrhexis with phacoemulsification and intraocular lens implantation, pars plana vitrectomy, and removal of IOFB in the left eye. Six years later, he revisited our clinic. On slit lamp examination, the corneal haziness had worsened, and the iris showed heterochromia resembling the spokes of a wheel in the left eye.

**Diagnosis:**

Ocular siderosis.

**Intervention:**

Anterior and posterior segment OCT was performed.

**Outcomes:**

The anterior segment OCT showed linear hyperreflectivity on the anterior corneal stroma just beneath the Bowman's layer. The posterior segment OCT showed inner retinal degeneration observed at the parafoveal area.

**Lessons:**

Ocular siderosis progression can happen after the removal of IOFB. The swept source OCT might be useful to assess the cornea and retina in ocular siderosis patient with corneal haziness.

## 1 Introduction

Ocular siderosis is characterized by degenerative changes secondary to retained, iron-containing, intraocular foreign body (IOFB).^[1]^ Clinical findings include, but are not limited to, rust-colored hue of the corneal stroma, iris heterochromia, pupillary mydriasis, secondary glaucoma, relative afferent pupillary defect, cataract, vitritis, retinal arteriolar narrowing, and sheathing.^[1,2]^ These ocular findings are well documented by slit lamp photography, angiography, and adaptive optics and usually improve after IOFB removal.^[3,4]^ However, very few studies report the optical coherence tomography (OCT) findings of ocular siderosis progression after IOFB removal. This case report presents the detailed OCT findings of progressive ocular siderosis after IOFB removal.

## 2 Case report

A 55-year-old Asian man presented to our clinic in January 2013 with the chief complaint of decreased vision in his left eye for 5 months, following an injury with an unknown foreign body while he was using a grass mower. He was first diagnosed with a left traumatic hyphema at the local medical center and was administered eye drops, with no improvement in visual acuity.

**Figure 1:**
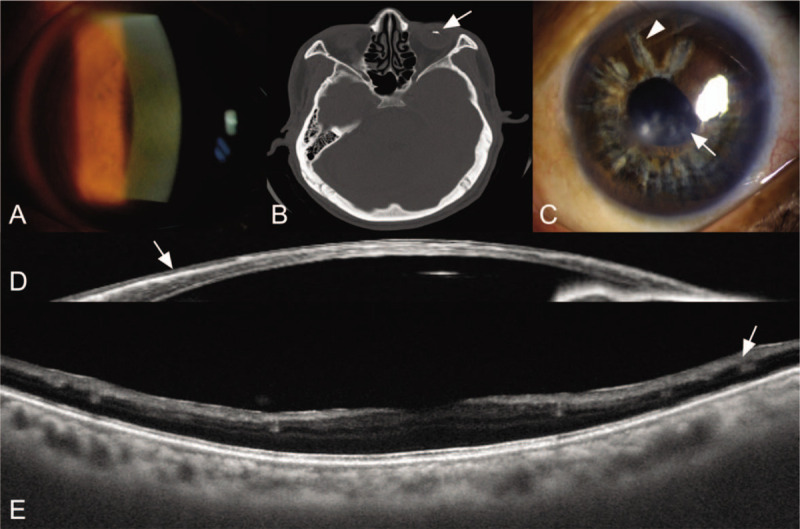
Ocular siderosis at initial visit and 6 years after removal of intraocular foreign body. (A) A slit lamp photograph at initial visit showing rust-colored hue visible on corneal stroma. (B) Orbital computed tomography showing intraocular foreign body (arrow) in the vitreous. (C) A slit lamp photograph at 6 years after removal of intraocular foreign body showing corneal haziness (arrow) and iris heterochromia (arrow head) resembling spokes of a wheel. (D) Anterior segment optical coherence tomography of ocular siderosis showing linear hyperreflectivity in the anterior corneal stroma (arrow). (E) Posterior segment optical coherence tomography of ocular siderosis showing degeneration of the inner retina (arrow).

At presentation, the left eye uncorrected visual acuity (UCVA) was 20/320 (measured with the Snellen chart), and intraocular pressure (IOP) was 7 mm Hg by applanation tonometry. The right eye had best corrected visual acuity (BCVA) of 20/20 and IOP of 11 mm Hg. On slit lamp examination of the left eye, the corneal stroma had a rust-colored hue, and the retina was not visible due to vitreous opacity (Fig. 1A). The examination of the right eye was unremarkable. An orbital computed tomography was ordered considering the possibility of left IOFB, which confirmed the presence of a vitreous IOFB (Fig. 1B). On the next day, we performed a continuous curvilinear capsulorrhexis with phacoemulsification and intraocular lens implantation, pars plana vitrectomy, and removal of the IOFB in the left eye. The IOFB was ferrous metal floating in the vitreous. The IOFB was retrieved in 1 piece using magnetic forceps. During the surgery, the diffuse inferior chorioretinal degeneration was observed. At the 1-month postsurgical follow-up, the left eye BCVA was 20/320, and the corneal and fundus findings remained unchanged.

Six years later, in June 2019, the left eye UCVA was 20/500 and IOP was 6 mmHg by applanation tonometry. On slit lamp examination, the corneal haziness had worsened, and the iris showed heterochromia resembling the spokes of a wheel (Fig. 1C). The fundus was not visible. Anterior and posterior segment OCT (DRI OCT-1; Topcon, Tokyo, Japan) was performed.

The anterior segment OCT showed linear hyperreflectivity on the anterior corneal stroma just beneath the Bowman's layer, and not as separate round particles (Fig. 1D). The swept-source OCT provided good visualization of the retina, which was difficult to assess by slit lamp examination. A definite hyperreflectivity was not seen in the retina, and the foveal region showed an intact microstructure. However, there was inner retinal degeneration observed at the parafoveal area (Fig. 1E). Informed written consent was obtained from the patient for publication of this case report and accompanying images.

## 3 Discussion

It is known that the progression of ocular siderosis is arrested by the removal of an IOFB.^[5]^ However, in this case report, we describe a rare progression of ocular siderosis even after removal of the iron-containing foreign body.

In our case, the OCT revealed anterior linear hyperreflectivity in the corneal stroma just beneath the Bowman's layer. It is known that siderosomes are found only within the keratocytes of the cornea.^[6]^ The high density of keratocytes with siderosomes in the anterior corneal stroma might explain the linear hyperreflectivity on the anterior segment OCT. We also found the inner retinal degeneration, which might indicate decreased perfusion. This is consistent with a previous study on IOFB, which demonstrated that iron toxicity produced more damage to the inner retina.^[7]^ An IOFB not embedded in the retina can also be related to the intact outer retina. Weiss et al^[8]^ mentioned that ocular siderosis could be reversible and could result in an up to 40% decrease in electroretinogram (ERG) b-wave amplitude. We have not performed ERG in this case; however, we propose that the long period that the IOFB was retained in the vitreous may have resulted in retinal damage with a > 40% decrease in ERG b-wave amplitude, and thus progressive changes might have occured from the remaining iron particles in the retina after the IOFB removal.^[9]^ In the current case, OCT facilitated good visualization of structural changes of ocular siderosis progression. The OCT might be useful to assess the cornea and retina in patients with ocular siderosis and corneal haziness.

## 4 Conclusion

To conclude, we have reported a case of OCT findings of ocular siderosis that progressed even after IOFB removal. We suggest that the disseminated iron particles from the previously retained IOFB can remain in the ocular tissues after its removal, resulting in continued toxicity beyond the initial macrophagic activities. This case also highlights that late removal of IOFB may not prevent further siderotic changes and that urgent removal of an IOFB is needed to prevent continuous siderotic aggravation as well as infection.

## Author contributions

**Conceptualization:** Yu Cheol Kim.

**Data curation:** Yu Cheol Kim, You Hyun Lee.

**Methodology:** Yu Cheol Kim, You Hyun Lee.

**Visualization:** Yu Cheol Kim, You Hyun Lee.

**Writing – original draft:** You Hyun Lee.

**Writing – review and editing:** Yu Cheol Kim.
